# Effect of airway acidosis and alkalosis on airway vascular smooth muscle responsiveness to albuterol

**DOI:** 10.1186/s40360-015-0008-y

**Published:** 2015-04-02

**Authors:** Jose E Cancado, Eliana S Mendes, Johana Arana, Gabor Horvath, Maria E Monzon, Matthias Salathe, Adam Wanner

**Affiliations:** Division of Pulmonary, Allergy, Critical Care and Sleep Medicine, University of Miami School of Medicine, Miami, FL 33136 USA; Department of Pulmonology, Semmelweis University School of Medicine, Budapest, Hungary

**Keywords:** Airway surface liquid pH, Airway blood flow, Respiratory alkalosis, Respiratory acidosis, Albuterol

## Abstract

**Background:**

*In vitro* and animal experiments have shown that the transport and signaling of β_2_-adrenergic agonists are pH-sensitive. Inhaled albuterol, a hydrophilic β_2_-adrenergic agonist, is widely used for the treatment of obstructive airway diseases. Acute exacerbations of obstructive airway diseases can be associated with changes in ventilation leading to either respiratory acidosis or alkalosis thereby affecting albuterol responsiveness in the airway. The purpose of this study was to determine if airway pH has an effect on albuterol-induced vasodilation in the airway.

**Methods:**

Ten healthy volunteers performed the following respiratory maneuvers: quiet breathing, hypocapnic hyperventilation, hypercapnic hyperventilation, and eucapnic hyperventilation (to dissociate the effect of pH from the effect of ventilation). During these breathing maneuvers, exhaled breath condensate (EBC) pH and airway blood flow response to inhaled albuterol (ΔQ̇_aw_) were assessed.

**Results:**

Mean ± SE EBC pH (units) and ΔQ̇_aw_ (μl.min^-1^.mL^-1^) were 6.4 ± 0.1 and 16.8 ± 1.9 during quiet breathing, 6.3 ± 0.1 and 14.5 ± 2.4 during eucapnic hyperventilation, 6.6 ± 0.2 and -0.2 ± 1.8 during hypocapnic hyperventilation (p = 0.02 and <0.01 vs. quiet breathing), and 5.9 ± 0.1 and 2.0 ± 1.5 during hypercapnic hyperventilation (p = 0.02 and <0.02 vs quiet breathing).

**Conclusions:**

Albuterol responsiveness in the airway as assessed by ΔQ̇_aw_ is pH sensitive. The breathing maneuver associated with decreased and increased EBC pH both resulted in a decreased responsiveness independent of the level of ventilation. These findings suggest an attenuated response to hydrophilic β_2_-adrenergic agonists during airway disease exacerbations associated with changes in pH.

**Trial registration:**

Registered at clinicaltrials.gov: NCT01216748.

## Background

*In vitro* and animal experiments have shown that transport of and signaling by β_2_-adrenergic agonist are pH-sensitive. At acidic pH, the transport of β_2_-adrenergic agonists across the airway epithelium is decreased [1], β_2_-adrenergic receptor function is impaired [2,3], endothelial function is diminished [4-6], and systemic vascular smooth muscle tone is increased [7]. Conversely, epithelial β_2_-adrenergic agonist transport is increased at alkaline pH [1]. The effects of alkalosis on β_2_-adrenergic receptor function, endothelial function and systemic vascular smooth muscle tone are less clear, with studies showing minimal or no changes in β_2_-adrenergic signaling [5], but an increase in vascular smooth muscle tone [7].

Inhaled albuterol, a hydrophilic β_2_-adrenergic agonist, is widely used for the treatment of obstructive airway disease. Acute exacerbations of obstructive airway diseases can be associated with changes in ventilation leading to either respiratory acidosis or alkalosis. The resulting changes in airway pH could have an effect on albuterol responsiveness. We therefore sought to test the hypothesis that the magnitude of vasodilation in the airway caused by inhaled albuterol could be altered by changes in airway pH. To investigate this possibility, we determined the effect of airway surface pH on airway blood flow (Q̇_aw_) responsiveness to inhaled albuterol in healthy subjects by manipulating airway pH through ventilatory maneuvers. Healthy subjects were chosen because the required respiratory maneuvers would be difficult to impose on patients with airflow obstruction. Q̇aw was chosen as a “biomarker” of albuterol responsiveness because airflow responses would only be marginally sensitive to albuterol in healthy subjects.

## Methods

### Subjects

Ten healthy lifetime non-smokers participated in the study. The exclusion criteria were as follows: *1*) a physician diagnosis of cardiovascular or pulmonary disease; *2)* the use of cardiovascular or airway medication; *3*) a body mass index >30; and *4*) a forced expiratory volume in 1 second (FEV_1_) < 80% of predicted and FEV_1_-to-forced vital capacity ratio < 0.7. All subjects had been free of an acute respiratory infection for at least 4 weeks before beginning the study, and no subject had an acute respiratory infection during the study. The study was approved by the Western Institutional Review Board and by the Human Subjects Research Office at the University of Miami. A signed informed consent was obtained from the subjects. The study is registered at clinicaltrials.gov: NCT01216748.

### Measurements

#### Airway blood flow (Q̇aw)

A previously validated soluble inert gas uptake method was used to measure Q̇_aw_ [8,9]. The subjects first inhaled room air to total lung capacity. After exhaling 500 mL, they rapidly re-inhaled the same volume of a pre-mixed gas consisting of 10% dimethylether (DME), balance nitrogen. After a predetermined breathhold time, the subjects then exhaled through a critical flow orifice to standardize the expiratory flow. During the entire maneuver, the instantaneous concentrations of DME and nitrogen were measured at the airway opening with a mass spectrometer (Perkin-Elmer; Pomona, CA). The maneuver was performed with two breathhold times each of 5 and 15 sec in random order. The DME concentration (F_DME_) at the end of phase 1 of the nitrogen wash-in curve (defining a virtual anatomical dead space, V_D_) was obtained. The difference in F_DME_ between the two breathhold times (∆F_DME_) multiplied by V_D_ was used to calculate DME uptake (V̇_DME_) over the intervening 10 sec. From V̇_DME_, the mean DME concentration between the two breathholds (F_mDME_) and the solubility coefficient for DME in blood and tissue (α), was calculated using the Fick principle (Q̇_aw_ = V̇_DME_/(α•F_mDME_). Q̇_aw_ was normalized for V_D_; therefore, V_D_ cancels out and wasn’t measured. Q̇_aw_ was expressed as μl.min^-1^.mL^-1^, where μl.min^-1^ reflects blood flow and mL reflects the virtual anatomical deadspace. At each Q̇_aw_ determination, data from two 5 sec and two 15 sec breathholds were analyzed. A Q̇_aw_ determination took less than 5 min.

Blood pressure and arterial oxygen saturation (SaO_2_) by pulse-oximetry were monitored at each measurement point. Mean systemic arterial pressure (perfusion pressure for airway blood flow) was calculated as diastolic pressure plus 1/3 pulse pressure.

#### Spirometry

For spirometry (Forced Expired Volume in one second/FEV1, Forced Vital Capacity/FVC, FEV_1_/FVC), a Koko spirometer was used (Ferraris Respiratory, Louisville, CO). The tracing with the highest FVC of three forced vital capacity maneuvers was analyzed. Predicted normal values were taken from Crapo et al [10]. The values were expressed in absolute values and percent of predicted.

Exhaled Breath Condensate (EBC) pH was obtained as recommended by an American Thoracic Society/European Respiratory Society task force [11]. The EBC samples were collected with the condenser temperatures close to 0°C. We determined EBC pH immediately following sample collection without argon purging [12], using a Thermo Orion 3 Star pH Meter and Micro pH Electrode (Thermo Scientific Orion Inc., Carlsbad, CA). During the different breathing maneuvers, EBC samples were collected by directing the subject’s exhaled breath into a pre-cooled (-10°C) tube for 5 min, using the disposable R-tubes® from Respiratory Research System (Charlottesville, VA). Over this period of time, approximately 0.5-1 mL of condensate was collected. For further standardization, the subjects were not allowed to drink or eat for at least one hour before the EBC samples were collected [13,14].

#### Ventilation

Compressed air was lead through a calibrated airflow regulator (Dakota Instruments, Orangeburg, NY) and an anesthesia bag to a one-way valve at the mouthpiece. During the ventilatory maneuvers, the airflow was adjusted to keep the anesthesia bag from collapsing or overinflating until a steady state was reached [15]. The airflow was read at that point and expressed as l.min^-1^. The system had a deadspace of 100 mL between the mouthpiece and the valve separating inspiration from expiration. Subjects wore a nose clip for all measurements.

### Respiratory maneuvers

Different respiratory maneuvers were used to change airway pH as reflected by EBC pH.

The same measurements were made in all subjects during quiet breathing, hypercapnic hyperventilation, hypocapnic hyperventilation and eucapnic hyperventilation. To induce hypercapnic hyperventilation, we employed a modification of a previously described procedure [15]. While monitoring S_a_O_2_ using pulse oximetry and end-tidal CO_2_ by mass-spectrometry (Perkin-Elmer, Pomona, CA) on a breath by breath basis, CO_2_ was bled into the inspired air to achieve an end-tidal pCO_2_ of at least 55 mmHg, expected to result in a decrease in systemic pH of about 0.1 pH units. For hypocapnic hyperventilation, the subjects were instructed to breathe fast and deep until their end-tidal pCO_2_ fell to 30 mmHg, corresponding to a systemic pH increase of about 0.1 pH units. For eucapnic hyperventilation, the subjects were instructed to increase their ventilation to the highest level of ventilation recorded in the previous two hyperventilation maneuvers, while CO_2_ was bled into the inspired air to maintain end-tidal pCO_2_ at 40 mmHg. This maneuver was used to separate the effect of ventilation from the effect of pH on albuterol responsiveness. The same mouthpiece set-up was used for the measurement of Q̇_aw_, EBC pH, and ventilation.

### Protocol

The subjects were instructed to abstain from ingesting alcoholic beverages the night before each study day and not to ingest caffeinated drinks for at least 12 hours before the study. The subjects were also instructed not to use phosphodiesterase type 5 inhibitors for 12 hours before coming to the laboratory.

There were 6 visit days. On day 1, informed consent was obtained and the subjects underwent a physical examination to ensure good general health. In females, a urine pregnancy test was performed to rule out current pregnancy. Then, spirometry was performed to ensure normal lung function. For technical reasons, EBC pH, Q̇_aw_ responses to albuterol and the level of ventilation could not be assessed simultaneously during the breathing maneuvers. Therefore, these parameters were measured during different breathing maneuvers on different days in random order (quiet breathing, hypercapnic hyperventilation, hypocapnic hyperventilation and eucapnic hyperventilation_._

#### Exhaled breath condensate collection

For each respiratory maneuver, the subjects breathed at the respective ventilatory level for 2 minutes followed by a 5 minutes EBC collection while maintaining the same breathing pattern.

#### Determination of ventilation

This was done during the different respiratory maneuvers as described for the EBC collection. Ventilation was measured during the 5 min steady state period.

#### Q̇_aw_ response to albuterol

This was done during the four breathing protocols as described above. During the 5 min steady state breathing period, Q̇_aw_ was first measured with a short break in the breathing maneuver. After resuming the designated breathing maneuver, the subjects inhaled albuterol (180 μg) delivered by a metered dose inhaler using a holding chamber during a brief interruption of the breathing maneuver. The subjects then continued to perform the prescribed respiratory maneuver for another 5 min. Q̇_aw_ was again measured 15 min after drug administration during quiet breathing. Albuterol responsiveness was expressed as the difference between pre-and post albuterol Q̇_aw_ (ΔQ̇_aw_).

### Statistical analysis

Values are presented as mean ± standard error (SE). Differences between the groups were analyzed by a non-parametric Kruskal-Wallis ANOVA test followed, when significant, by the Mann-Whitney *U* test for comparisons between groups. Values were expressed as mean ± SE and a p value less than 0.05 was accepted as a statistically significant difference. All statistics were analyzed with SPSS software (Statistical Product and Services Solutions, version 18.0; SPSS Inc., Chicago, IL).

## Results

The demographics and baseline characteristics of study participants are shown in Table 1, consistent with good cardiovascular and respiratory health. All subjects completed the protocol.

**Table 1:** **Demographics and baseline characteristics of study participants (visit 1)**

	**Subjects**
N	10
Mean age (range), yr	37 ± 8 (24-53)
Sex (M/F)	3 / 7
Heart rate, beats/min	65 ± 12
Systolic BP, mmHg	106 ± 5
Diastolic BP, mmHg	68 ± 4
SAT O_2_	99 ± 1
FEV_1_, liters	3.26 ± 0.61
FEV_1_, %predicted	104 ± 1

Values are mean ± SE.

N = number of subjects; M, male; F, female; BP, blood pressure; SAT O_2_, arterial oxygen saturation measured by pulse oximetry; FEV_1_, forced expiratory volume in 1 second.

### Ventilation and EBC pH

The levels of ventilation at the time of albuterol administration during the four respiratory maneuvers are shown in Table 2. Hypercapnia and hypocapnia changed EBC pH, while eucapnic hyperventilation had no effect on EBC pH. Thus, it was possible to unlink the level of ventilation from the changes in EBC pH, which presumably is a reflection of airway surface liquid pH.

**Table 2:** **Ventilation and exhaled breath condensate pH during respiratory maneuvers**

**Challenges**	**V̇ (L** ^**.**^ **min** ^**-1**^ **)**	**EBC pH (units)**
Quiet breathing	14.4 ± 4.2	6.39 ± 0.14
Eucapnic hyperventilation	35.5 ± 3.4*	6.31 ± 0.08
Hypocapnic hyperventilation	35.2 ± 3.3*	6.59 ± 0.15**
Hypercapnic hyperventilation	24.4 ± 2.9*	5.88 ± 0.14**

V̇, ventilation.

EBC, exhaled breath condensate.

*p < 0.05 vs. quiet breathing.

**p < 0.02 vs. quiet breathing.

### Airway blood flow response to albuterol

Mean systemic blood pressure and oxygen saturation were not different at the Q̇_aw_ measurement points (baseline,pre-albuterol and post albuterol). The lack of changes in mean systemic blood pressure obviated the need to express the airway blood flow responses as airway blood flow conductance. Vasodilator responses therefore were reported as ΔQ̇_aw_.

**Figure 1:**
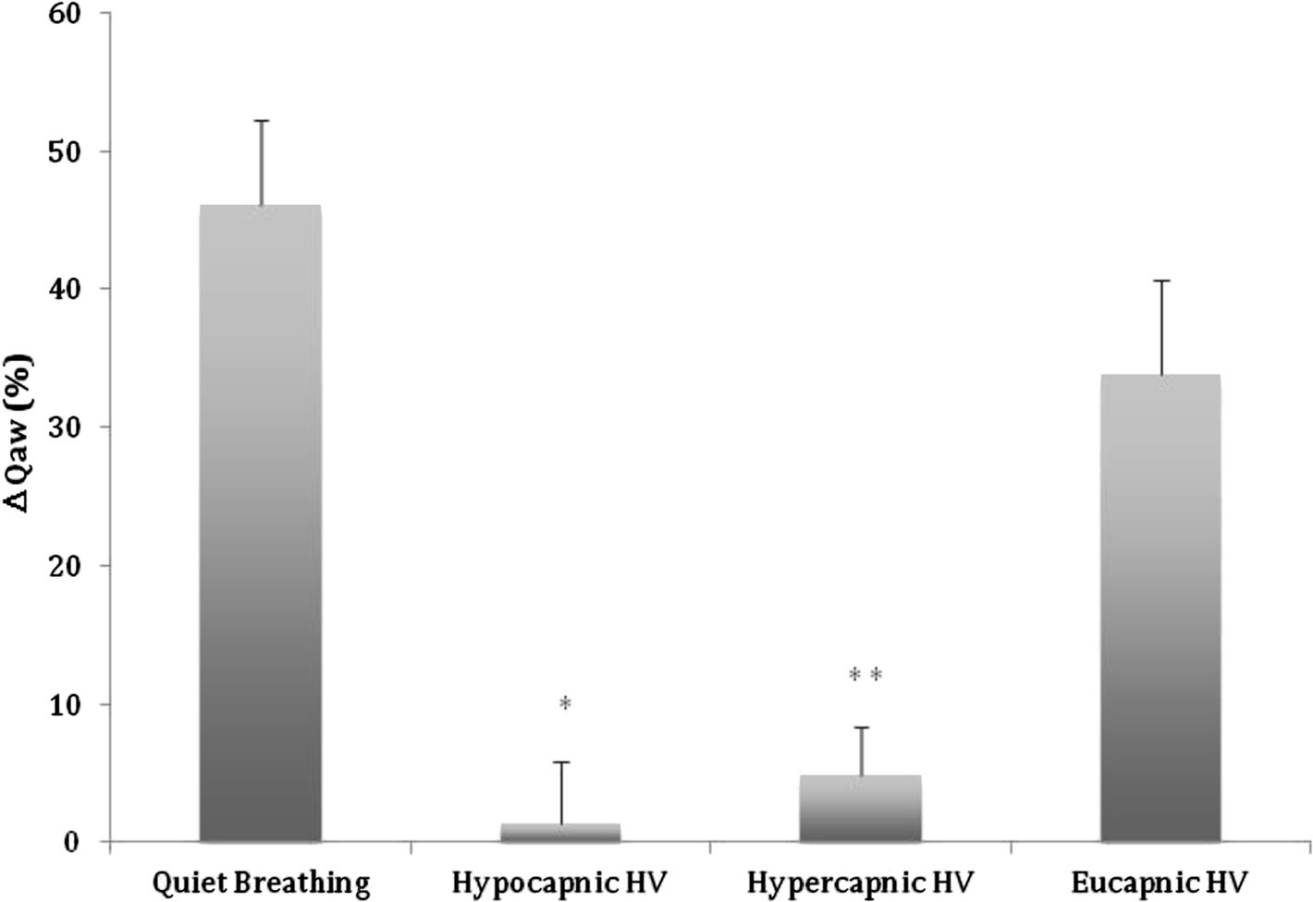
**Relative albuterol-induced changes in airway blood flow (ΔQ̇**_**aw**_ **) during four breathing maneuvers.** Values are mean ± SE. *p < 0.01 and ** p < 0.02 vs quiet breathing and eucapnic hyperventilation.

Baseline mean Q̇_aw_ values were similar before the four breathing maneuvers and remained unchanged during the subsequent breathing maneuvers as reflected by the pre-albuterol values (Table 3). All subjects had similar albuterol response to the different breathing maneuvers^.^ Albuterol increased mean Q̇_aw_ significantly, by 46.2 and 33.8% 15 min post drug inhalation during quiet breathing and eucapnic hyperventilation, respectively (Table 3, Figure 1). In contrast, albuterol had no effect on mean Q̇_aw_ during hypercapnic hyperventilation (4.9%) or hypocapnic hyperventilation (-1.3%) maneuvers, which were associated with a decrease or increase in EBC pH.

**Table 3:** **Effects of respiratory maneuvers on airway blood flow (Q̇**_**aw**_ **)**

		**Pre-maneuver Q̇**_**aw**_	**Q̇**_**aw**_ **during steady state**	**Q̇**_**aw**_ **15 min post albuterol**
Quiet breathing	Mean ± SE	38.1 ± 1.4	37.2 ± 1.5	53.7 ± 2.1*
	Median	37.7	35.7	54.3
	25% quartile	33.8	34.1	46.9
	75%quartile	42.1	41.9	58.7
Eucapnic hyperventilation	Mean ± SE	41.8 ± 4.3	42.6 ± 4.3	56.4 ± 4.0*
	Median	40.7	42.7	49.7
	25% quartile	34.8	34.8	46.7
	75% quartile	48.8	48.8	61.7
Hypocapnic hyperventilation	Mean ± SE	42.9 ± 2.8	45.2 ± 3.4	45.1 ± 2.9
	Median	40.1	42.2	43.7
	25% quartile	37.2	35.0	38.8
	75% quartile	49.1	49.7	54.1
Hypercapnic hyperventilation	Mean ± SE	42.1 ± 2.4	44.8 ± 3.8	46.7 ± 4.0
	Median	37.5	44.7	43.7
	25% quartile	35.5	34.1	38.4
	75% quartile	46.2	57.1	52.2

Q̇_aw_ is expressed in μl.min^-1^.mL^-1^ . * p < 0.05 vs. steady state.

## Discussion

The purpose of this study was to determine if respiratory acidosis and alkalosis have an effect on the physiological response to inhaled albuterol in airway tissue and if the effect is related to the ventilation-associated changes in airway pH as reflected by exhaled breath condensate (EBC) pH. In order to demonstrate the role of pH in the observed changes in albuterol responsiveness associated with respiratory acidosis and alkalosis, it was necessary to unlink the changes in EBC pH from the changes in ventilation. This was done by comparing quiet breathing with eucapnic hyperventilation, where ventilation changes while pH is kept constant. Albuterol responsiveness was the same during the two maneuvers, suggesting that hyperventilation per se did not alter albuterol responsiveness. Likewise, the preserved albuterol responsiveness during eucapnic hyperventilation ruled out the possibility that cooling and drying of the airway could have been the cause of the blunted albuterol responsiveness during respiratory alkalosis and acidosis. Eucapnic hyperventilation was investigated last in order to be able to reproduce the highest level of ventilation achieved in any of the other maneuvers. We therefore are confident that ventilation per se had no effect on albuterol responsiveness. The level of ventilation during quiet breathing was higher than one would have expected in healthy subjects at rest (mean 14.4 L^.^min^-1^). It has previously been reported that wearing a nose clip and breathing through a mouthpiece increases tidal volume and minute ventilation [16]. In addition, the breathing setup we used for our study included a 100 mL deadspace, another stimulus for increasing tidal volume and respiratory rate.

In our study, the intended target of albuterol was airway vascular smooth muscle contained in the airway wall. Since the different respiratory maneuvers by themselves had no effect on Q̇_aw_ we were able to assess the effect of respiratory acidosis and alkalosis on albuterol responsiveness. In some systemic vascular beds, hypercapnic acidosis causes relaxation and hypocapnic alkalosis causes constriction, resulting in corresponding blood flow changes [5]. The airway circulation appears not to be subject to this regulation at least in the range of pCO_2_ changes seen in the present study in which changes in pH had no effect on Q̇_aw_; however, they affected albuterol responsiveness. We allowed 5 min for albuterol absorption during the four breathing maneuvers, and measured Q̇_aw_ 15 min after drug inhalation. This was done because in previous studies we found that the maximum response typically occurs after 15 min while a vasodilator response to inhaled albuterol is already seen after 5 min [17].

We found that both airway alkalosis and acidosis attenuated albuterol responsiveness. The pH-sensitivity of albuterol responsiveness could have been related to a combination of several factors, including absorption and transport of albuterol from the airway surface to the airway vascular smooth muscle, β_2_-adrenergic receptor function, vascular endothelial function or vascular smooth muscle responsiveness*. In vitro* and animal experiments suggest that all of these functions can be pH-dependent.

### Acidosis

The majority of the currently used β_2_-adrenergic bronchodilators, including albuterol, cannot freely diffuse across the epithelial cell membrane because they are hydrophilic and carry a transient or permanent positive charge at physiological pH. Thus, the epithelium of the airway becomes a barrier to these agents, requiring cellular or paracellular transport across the epithelial lining of the airway to reach their intended target tissues including airway vascular smooth muscle. We have previously demonstrated the existence of an organic cation transport machinery in the human airway epithelium and showed that this process is largely mediated by the organic cation/carnitine transporter OCTN2, which is likely involved in the delivery of inhaled hydrophilic cationic bronchodilators to the airway tissue [1]. We showed that cationic drug uptake is pH dependent, with about 3-fold lower rates at an acidic pH (5.7) than alkaline pH (8.2). This mechanism could have been fully or partially responsible for the blunted albuterol responsiveness during respiratory acidosis associated with a decreased airway surface liquid pH. We have also shown that albuterol crosses the airway epithelium via the paracellular route [18]. The paracellular pathway can also mediate pH-dependent permeability to pH-dependent changes in negative charges.

It has also been reported that acidosis can cause rapid desensitization and uncoupling of β_2_-adrenergic receptors [2], possibly leading to albuterol unresponsiveness as seen in the present investigation.

Albuterol-induced vasodilation is endothelium-dependent, involving endothelial relaxant factors including nitric oxide [19]. Although the observations on the effects of intracellular and extracellular acidosis and pCO_2_ on endothelial function have not been consistent, the majority of studies have shown that acidosis can impair endothelial function [4-6]. It is likely that airway surface liquid pH is a reflection of extracellular pH, but changes in both extracellular and intracellular pH have been implicated in the effect of acidosis on endothelial function. Thus, endothelial dysfunction could have had a role in the blunted albuterol responsiveness in our study. Finally, airway vascular smooth muscle function could be directly affected by acidosis. In particular, acidosis can lead to smooth muscle cell hyperpolarization, which in turn could attenuate albuterol-induced vasodilation [5].

### Alkalosis

Respiratory alkalosis also attenuated albuterol-induced vasodilation in our study. *In vitro*, alkalosis increases the transport of organic cations such as albuterol across the airway epithelium via the transcellular and paracellular routes [18]. Alkalosis may also increase β_2_-adrenergic receptor ligand binding [3]. Finally, alkalosis has been shown to cause endothelium-dependent vasodilation without altering endothelial nitric oxide synthase function [5]. All of these actions would be expected to potentiate inhaled albuterol-induced vasodilation. The mechanistic explanation for our observation that respiratory alkalosis has the same attenuating effect on albuterol responsiveness as acidosis remains unclear at this time.

## Conclusions

Patients with airway disease are likely to have highly variable airway surface liquid pH and adrenergic airway smooth muscle responsiveness [20-23]. Therefore, we decided to investigate the pH dependence of β_2_-adrenergic responsiveness as a marker of albuterol responsiveness in healthy subjects with normal β_2_-adrenergic smooth muscle responsiveness in whom the airway surface liquid pH can be artificially manipulated. From a clinical perspective, airway smooth muscle would have been a more meaningful airway wall target to assess responsiveness to inhaled albuterol. However, healthy subjects do not have an increased airway smooth muscle tone and responses to albuterol would have been too small to study the effects of respiratory acidosis and alkalosis. We chose not to include patients with asthma or COPD in the investigation because they have a blunted airway blood flow response to albuterol due to endothelial dysfunction [18], and because measuring airflow responses by pulmonary function testing would have been technically difficult under the experimental conditions of the study.

Our *in vivo* observation showed that both respiratory acidosis and alkalosis blunt albuterol responsiveness in the airway wall, although it is not known whether the effect is driven by intracellular or extracellular pH or pCO_2_ and which of the above-mentioned mechanisms may be involved. In this study we found that albuterol responsiveness as assessed by Q̇_aw_ in the airway is blunted by acidosis and alkalosis, using Q̇_aw_ as a bioassay. It remains to be shown whether the clinical benefits of inhaled albuterol, i.e., bronchodilation may be less than expected during acute respiratory acidosis and alkalosis, which can be associated with exacerbations of asthma and COPD.
